# Thickness-modulated metal-to-semiconductor transformation in a transition metal dichalcogenide

**DOI:** 10.1038/s41467-018-03436-0

**Published:** 2018-03-02

**Authors:** Alberto Ciarrocchi, Ahmet Avsar, Dmitry Ovchinnikov, Andras Kis

**Affiliations:** 1 Electrical Engineering Institute, École Polytechnique Fédérale de Lausanne (EPFL), 1015 Lausanne, Switzerland; 2 Institute of Materials Science and Engineering, École Polytechnique Fédérale de Lausanne (EPFL), 1015 Lausanne, Switzerland

## Abstract

The possibility of tailoring physical properties by changing the number of layers in van der Waals crystals is one of the driving forces behind the emergence of two-dimensional materials. One example is bulk MoS_2_, which changes from an indirect gap semiconductor to a direct bandgap semiconductor in the monolayer form. Here, we show a much bigger tuning range with a complete switching from a metal to a semiconductor in atomically thin PtSe_2_ as its thickness is reduced. Crystals with a thickness of ~13 nm show metallic behavior with a contact resistance as low as 70 Ω·µm. As they are thinned down to 2.5 nm and below, we observe semiconducting behavior. In such thin crystals, we demonstrate ambipolar transport with a bandgap smaller than 2.2 eV and an on/off ratio of ~10^5^. Our results demonstrate that PtSe_2_ possesses an unusual behavior among 2D materials, enabling novel applications in nano and optoelectronics.

## Abstract (web summary)

The electronic band structure of van der Waals crystals is strongly sensitive to the number of layers. Here, the authors observe a thickness-dependent metal-to-semiconductor transition in layered PtSe_2_ by means of electrical transport measurements.

## Introduction

Two-dimensional crystalline materials such as graphene, transition metal dichalcogenides (TMDCs), and black phosphorus (BP) have attracted considerable attention in recent years, due to a set of properties that make them relevant for both fundamental science and technological applications^1–3^. Due to the interaction between atomic layers, these materials exhibit dramatically different properties compared to their bulk forms^4–9^. While the field was opened with the discovery of graphene^10^, the presence of a bandgap in TMDCs has made this class of materials appealing for applications in electronics^11^. A key enabler in the development of two-dimensional electronics is the ability to control the properties of the constituent materials, in order to modulate or complement their natural characteristics. Among these, the bandgap is a crucial one, governing both optical and transport properties. For example, a configurable bandgap can be used to tune the adsorption spectrum to specific wavelengths^12^, while the bandgap is also an important factor determining the on-state current and on/off ratio in field-effect devices. Due to their atomic scale thickness, van der Waals materials are susceptible to several methods of bandgap modulation, involving strain, pressure, chemical or substitutional doping, and application of electrical fields^13–22^. However, it is difficult to implement most of these methods in devices without increasing their complexity. A stable and simple approach to bandgap engineering of van der Waals materials involves merely changing the number of layers in the crystal. Apart from exfoliation, this method could be exploited on a large scale by engineering the growth process or by selectively thinning the material after deposition. For example, in the case of MoS_2_ the 1.29 eV indirect bandgap in bulk form becomes a direct bandgap with a 1.8 eV optical resonance in monolayers^4–7^. Even more striking is the case of BP, where the bandgap modulation ranges from 0.35 eV in bulk to 1.73 eV in a monolayer^23–25^. However, in these systems we do not see a fundamental change of their semiconducting nature; we only see a change in the magnitude and type (direct vs. indirect) of the bandgap.

A much wider, qualitative change in the nature of electrical conductivity would extend the reach of two-dimensional materials further. It would, for example, allow the same material to be used both as an interconnect and as the semiconducting channel, greatly simplifying the realization of electronic circuits based on two-dimensional materials, as already proposed for PdS_2_ and hydrogenated graphene^26,27^. One material that could offer this is PtSe_2_, a group VIIIc transition metal dichalcogenide. It shares the same layered structure with other MX_2_ compounds, with each layer of the crystal made of a two-dimensional close-packed array of Pt atoms sandwiched between Se atoms in a 1T structure^28^. The electronic properties of bulk PtSe_2_ have been controversial, with experiments suggesting both a semiconductor^29^ and a semimetal^30,31^. More recently, it has been shown that in its bulk form PtSe_2_ is expected to be a semimetal^28,32^, with the opening of a bandgap in monolayers^33–35^. Recent studies have also shown that this material can be grown, and that it exhibits appreciable transport properties and interesting spin physics, motivating further studies of its transport properties^34,36,37^.

Here, we fabricate electronic devices based on PtSe_2_ and study electrical transport properties of crystals ranging in thickness from 14 to 2 nm. They show a transition from a metal to a semiconductor for thicknesses lower than 2.5 nm. Metallic samples show low contact resistance, while in semiconducting samples we observe ambipolar transport and estimate the size of the bandgap by employing electric-double-layer transistors (EDLTs).

## Results

### Device characterization

**Fig. 1:**
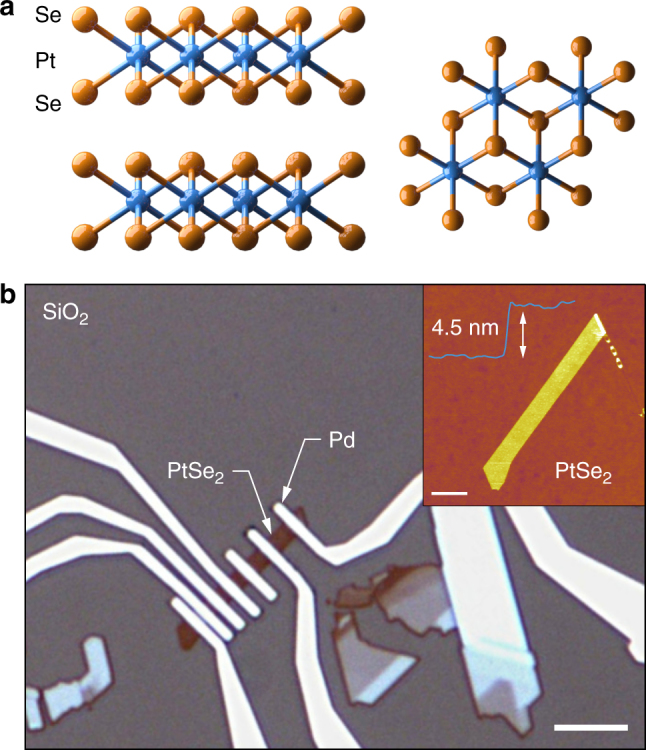
PtSe_2_ structure and devices. **a** PtSe_2_ layered crystal structure showing two layers from side and top view. **b** Example of PtSe_2_ crystal before (inset, AFM image, scale bar is 2 µm) and after evaporation of Pd contacts (optical image). Scale bar is 5 µm

**Fig. 2:**
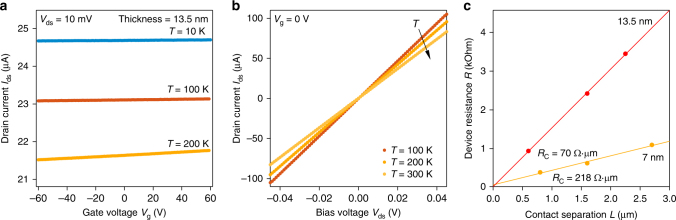
Electrical transport characterization of 13.5 nm thick PtSe_2_. **a** Gating characteristics showing weak current modulation at different temperatures. **b** *I*_ds_ − *V*_ds_ curves acquired at different temperatures. **c** Contact resistance extraction using the transfer length method for the 13.5 and 7 nm thick devices

Ultrathin PtSe_2_ crystals with 1T-phase (Fig. 1a) were obtained by mechanical exfoliation on doped Si wafers covered with 270 nm of SiO_2_. AFM imaging was used to precisely measure the thickness of the resulting crystals, followed by evaporation of Pd contacts to obtain back-gated devices such as the one shown in Fig. 1b. Depending on the crystal size and geometry, simple two-terminal devices, as well as devices with variable electrode spacing needed for contact resistance determination using the transfer length method were prepared. Transfer characteristics of the devices were measured in vacuum at different temperatures between 300 and 10 K (see methods). We first discuss the case of the thickest sample (13.5 nm) we characterized, which is representative of our observations for samples thicker than 7 nm. As expected from theoretical predictions of bulk PtSe_2_ being a semimetal, the device shows a metallic behavior with almost negligible n-type current modulation by the back gate (d*I*_ds_/d*V*_g_~10^–10^ S) and a perfectly linear bias (*I*_ds_−*V*_ds_) dependence, as shown in Fig. 2a, b. We calculate a sheet resistance of 400 Ω/sq at 10 K, which increases with temperature. Using the transfer length method, we extract a contact resistance as low as 70 Ω·µm at 10 K (Fig. 2c), significantly lower than the lowest reported numbers for semiconducting TMDCs with phase-engineered^38^ or degenerately doped^39^ contacts, indicating that the metallic PtSe_2_ could be an interesting material for realizing contacts to two-dimensional semiconductors^40^.

**Fig. 3:**
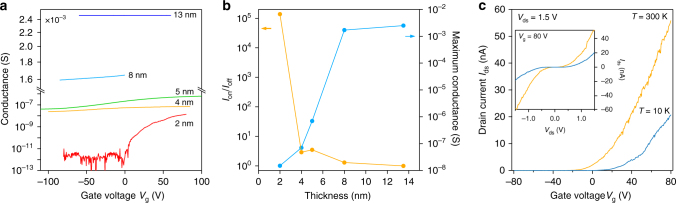
Thickness modulation of transport in PtSe_2_. **a** Conductance modulation as a function of gate voltage for different sample thicknesses. **b** Maximum conductance and on/off ratio as a function of sample thickness at 10 K. **c** *I*_ds_ − *V*_g_ curves recorded for the 2 nm thick device, showing n-type semiconducting behavior. Inset: *I*_ds_ − *V*_ds_ curves recorded for the same device

We now turn our attention to crystals thinner than 7 nm, measuring the conductance as a function of the back-gate voltage. In Fig. 3a, we present a summary of the conductance dependence on the back-gate voltage *V*_g_ measured at 10 K for devices with different thicknesses of PtSe_2_. Starting with an almost gate-independent conductance in bulk samples, indicating efficient screening of the gate voltage by the high density of charge carriers in the metallic sample, in samples with a thickness of 5 nm we clearly start to observe a significant non-linear modulation with the presence of a well-developed off-state and n-type conduction in the thinnest sample we measured (2 nm). This change in electrical transport properties is consistent with the presence of a finite number of states around the Fermi level in bulk PtSe_2_, which has been predicted by calculations^28,34,35^. The shrinking density of states with decreasing thickness results in a greater tunability of the conductance with the gate, up to the point where an appreciable bandgap is opened. This transition is summarized in Fig. 3b, where we observe a progressive decrease of the maximal conductance by more than five orders of magnitude compared to our thickest sample, from 2.5 to 15 nS in 2-nm-thick samples. This is accompanied by an increase in the relative current modulation that can be obtained, from ≈1 for the 13.5 nm thick sample to ≈10^5^ (2 nm). The semiconducting behavior in the 2-nm-thick sample can be more clearly seen in Fig. 3c. We measure the modulation of the drain current *I*_ds_ as a function of back-gate voltage *V*_g_. The device turns off even at room temperature, while the conductivity increases with the temperature (Fig. 3c, inset), as expected from a semiconductor. We have also made devices based on monolayer PtSe_2_ (see Supplementary Fig. 1), for which no conduction could be detected. This follows the observed trend of the reduction of conductivity with reduced thickness, making few layers the optimal thickness range for transistor-based applications, where a compromise between the high on-state current and on/off ratio are required.

### PtSe_2_ EDLT characterization and bandgap measurement

**Fig. 4:**
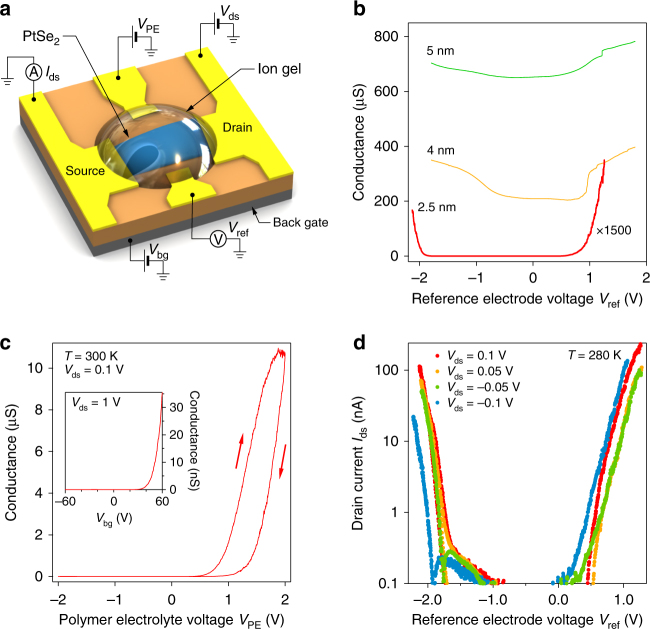
Devices with ion-gel dielectrics. **a** Device schematic. **b** Conductance as a function of electrostatically induced doping. **c** Device conductance for the device with 2.5 nm thick PtSe_2_ as a function of polyelectrolyte gate voltage *V*_PE_ at 300 K. **d** *I*_ds_ − *V*_ref_ dependence for the device with 2.5 nm thick PtSe_2_

To gain further insight into the behavior of PtSe_2_, we fabricate EDLTs based on the ([EMIM]-[TFSI]) ion gel^41,42^ and PtSe_2_ crystals of different thicknesses, with the device schematic shown in Fig. 4a. Because of the higher gate capacitance^41^, this approach allows us to explore electrical transport in a wider range of electrostatically induced doping, while in the same time we also benefit from a reduced barrier for charge injection at the contacts^12^. Figure 4b shows the conductance as a function of polymer electrolyte gate voltage for PtSe_2_ crystals in the 2.5–5 nm thickness range. In agreement with our observations on back-gated devices, the 4 and 5 nm thick samples show three orders of magnitude higher conductivity compared to the thinnest one, but a relative modulation of less than 50%. Together with the absence of an off-state in thicker crystals (also see Supplementary Fig. 2), we now observe that the modulation is indeed ambipolar. The presence of both electron and hole conduction branches allows us to rule out that the high conductivity we observe in thick PtSe_2_ is due to intrinsic doping.

We move to the characterization of the 2.5-nm-thick PtSe_2_ crystal, which was used to realize a two-terminal device. The inset of Fig. 4c shows the room-temperature conductance as a function of back-gate voltage before coating the device with the ion gel. Applying a drain-source bias of 0.1 V, we observe a threshold voltage of 1.5 V on the n-side for the PE gate, and can reach on-state conductance exceeding 10 µS. The on/off ratio is higher than 10^5^, and we estimate FE mobility around 3 cm^2^V^−1^s^−1^. For comparison (Fig. 4c, inset), the back-gated operation resulted in an on-state conductance of 36 nS, threshold voltage of 48 V, and a maximum relative modulation around 10^4^.

Since we already observe the presence of a bandgap in the sample, we subsequently cool it down to 280 K to be able to apply a higher gate voltage, thanks to the wider electrochemical window of the polymer electrolyte^12^. We assume the electrolyte capacitance at this temperature to be ~4 µF/cm^2^, from previous reports^41^. As can be seen from Fig. 4d, the device shows clear ambipolar behavior, with an on/off ratio around 10^5^ and subthreshold swings (SS) of 83 and 106 mV/dec for holes and electrons, respectively.

The large electrostatic capacitance in ionic and polymer electrolyte-gated FETs allows us to determine the bandgap^43–48^ of PtSe_2_ in the semiconducting thickness range. The prerequisite for this is ambipolar operation. The value of the bandgap can then be extracted from the difference between the threshold voltages for electron and hole branches ${\mathrm{\Delta }}V_{{\mathrm{th}}} = |V_{{\mathrm{th}}}^{\mathrm{e}} - V_{{\mathrm{th}}}^{\mathrm{h}}|$. Using a reference electrode to provide an accurate reading of the voltage drop across the gel *V*_ref_ (see methods), the energy $\mathrm{e}{\mathrm{\Delta }}V_{{\mathrm{th}}}$ is in an ideal case, equal to the energy needed to add an electron to the bottom of the conduction band and to create a hole at the top of the valence band. To apply this technique, we sweep the gate voltage at different source-drain bias levels and extrapolate from a linear fit the two threshold voltages at zero bias for better precision (see Supplementary Fig. 3). We obtain $V_{{\mathrm{th}}}^{\mathrm{e}} = 0.4{\ \mathrm{V}}$,$V_{{\mathrm{th}}}^{\mathrm{h}} = - 1.8{\ \mathrm{V}}$, which gives a bandgap value of $E_{\mathrm{G}} = 2.2 \pm 0.1$ eV (where the error is estimated from the fit at different *V*_ds_).

Theoretical calculations of the PtSe_2_ band structure predict the bandgap of monolayers to be between 1.2 and 2.1 eV^34,35^ with a significant reduction in bilayers. No calculations for larger thicknesses are available at this time. Interestingly, even if our sample is definitely thicker than a bilayer, it still shows a sizeable bandgap allowing efficient current modulation at room temperature. This difference between the theoretical calculations and our results for the 2.5-nm-thick PtSe_2_ could be explained by a possible underestimation of the bandgap in DFT calculations. Our samples could also be covered by a disordered “dead layer”, implying that the actual thickness of the active channel is lower than the one inferred by AFM measurements. These points require further future investigation. We would finally like to note that our measured value represents the upper limit for the actual bandgap, since it has been shown that all non-idealities in the EDLT operation such as trap states (see Supplementary Fig. 4) or contact resistance^46^ can lead to an overestimated bandgap. However, our measurements show that a significant bandgap is already present in few-layer PtSe_2_.

## Discussion

Our results on PtSe_2_ highlight a new type of behavior in an atomic scale TMDC material, with room temperature electrical transport characteristics which can be tuned from metallic to semiconducting, allowing both high electrical conductivity and efficient transistor operation to be realized in the same material. By implementing ohmic contacts and boron nitride encapsulation, it would be further possible to access the pristine properties of this material^49^, where theory predicts mobility of up to ~1890 cm^2^V^−1^s^−1^ at room temperature^50^. Considering the air-stable nature of PtSe_2_ even in the monolayer limit (see Supplementary Fig. 1), these observations open new possibilities for the facile realization of atomic scale circuitry based on thickness-modulated TMDCs.

## Methods

### Device fabrication

PtSe_2_ crystals were obtained by mechanical exfoliation from bulk crystals (HQ Graphene) onto a doped Si substrate with 270 nm of SiO_2_. The substrate was imaged using an optical microscope (Olympus BX51M) equipped with a color camera. The thickness of selected crystals was confirmed using AFM topography imaging (Asylum Research Cypher). Metallic contacts were prepared using e-beam lithography and e-beam evaporation of Pd (60 nm). For PE gating, special gate electrodes were designed with a surface area much larger than that of the PtSe_2_ channel, in order to maximize the gate efficiency.

### Measurements

Devices were characterized at different temperatures inside a Janis cryostat under high vacuum (10^–6^ mbar). Drain currents were measured using a Keithley Sourcemeter 2450, while a Keithley Sourcemeter 2400 was used for the solid and PE gates. The drain voltage was kept at 100 mV for most measurements, while the source was grounded. Voltage drop across the polymer electrolyte was measured using a Keithley Electrometer 6415.

### EDLT operation

For PE gating, we used an ion gel made from an ethyl acetate solution of the triblock copolymer PS-PMMA-PS and the ionic liquid 1-ethyl-3-methylimidazolium bis(trifluoromethylsulfonyl)imide ([EMIM][TFSI]). The devices were spin coated with the gel (6000 rpm, 1 min) in Ar atmosphere. The voltage drop across the polyelectrolyte was measured by using Pd reference electrodes close to the device, in order to be able to refer the measurements to the voltage effectively acting on the channel, as shown in previous works^43,47^. As can be seen from the *V*_ref_ − *V*_PE_ plot (Supplementary Fig. 5), the gate efficiency is around 60%. A small hysteresis can be observed in the dependence of *V*_ref_ on *V*_PE_, as well as from the *I*_ds_ − *V*_PE_ dependence. However, the *I*_ds_ versus *V*_ref_ curves are almost free of hysteresis. This confirms that the hysteretic behavior is due to the gate–PE interface, and not due to the device itself^46^. The measurements were repeated with two different reference electrodes for increased reliability, yielding almost indistinguishable results.

### Data availability

The data that support the findings of this study are available from the corresponding author on reasonable request.

## Electronic supplementary material

Supplementary Information

Peer Review File
