# Supplementary material for: Thickness-modulated metal-to-semiconductor transformation in a transition metal dichalcogenide
Source: Nat Commun. 2018 Mar 2;9:919. doi: 10.1038/s41467-018-03436-0 (PMC5834615; doi:10.1038/s41467-018-03436-0)
Supplement: Supplementary file 1 — Supplementary Information [file 41467_2018_3436_MOESM1_ESM.pdf]

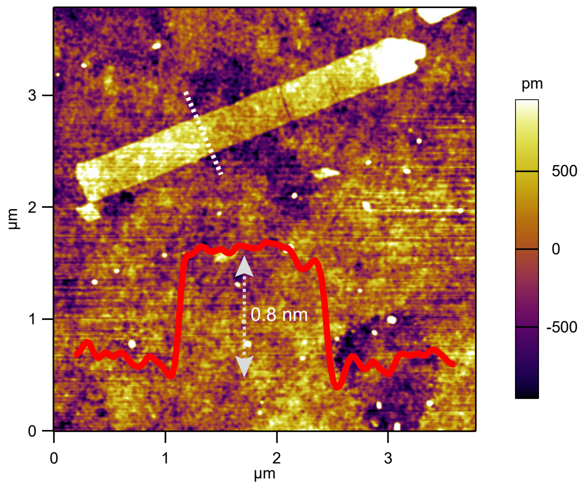

Supplementary figure 1. Monolayer $PtSe_2$. AFM image of a monolayer flake of $PtSe_2$ exfoliated on a SiO_2 substrate. The height profile along the dashed line is shown in red.

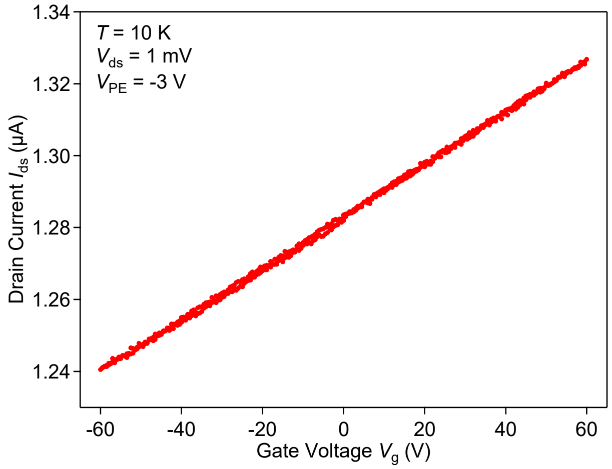

Supplementary Figure 2. EDLTs on thick $PtSe_2$ flakes. Drain current as a function of gate voltage at 10 K. The polymer electrolyte has been frozen at an applied gate voltage of -3 V, resulting in p-doping of the channel.

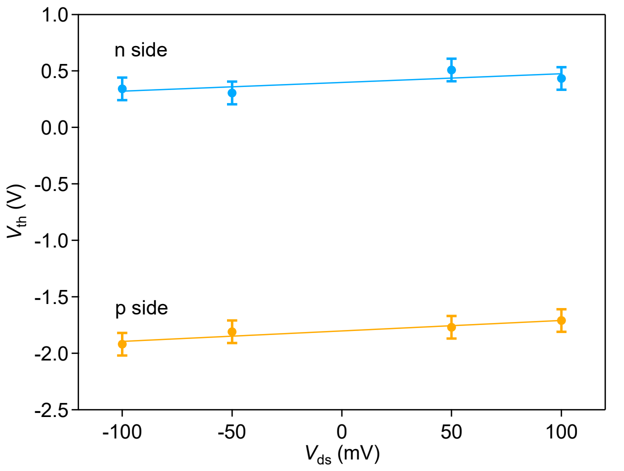

Supplementary Figure 3. Extraction of threshold voltage at zero bias. Threshold voltage values on the n-side (blue) and p-side (orange) for different drain-source biases and linear fit. Extracted thresholds at zero bias are 0.4 V and -1.8 V. The error bars are estimated from the standard deviation of the fitting used to extract the threshold voltage.

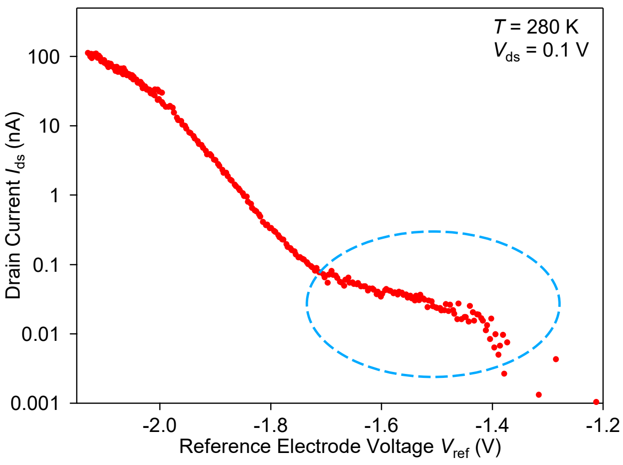

Supplementary Figure 4. Detail of transport curve at 280 K. Detail of subthreshold current on the p-side. In the dashed circle a small increase in current can be observed, characteristic of the presence of trap states in the gap.

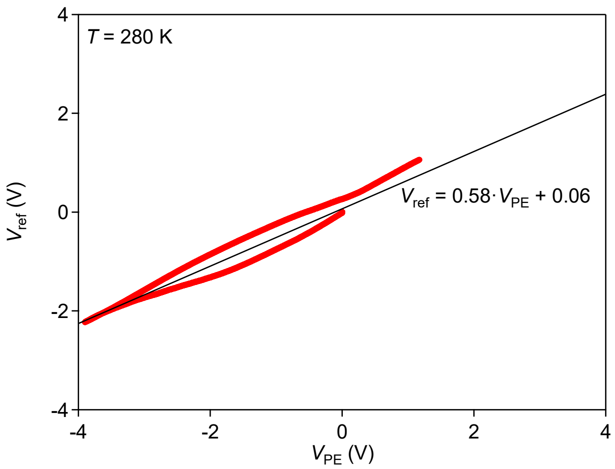

Supplementary Figure 5. Voltage drop across the polymer electrolyte. Measured reference voltage as a function of the applied gate voltage at 280K. From a linear fit of the data a 60% gate efficiency is extracted.
