# Supplementary material for: Thickness-modulated metal-to-semiconductor transformation in a transition metal dichalcogenide
Source: Nat Commun. 2018 Mar 2;9:919. doi: 10.1038/s41467-018-03436-0 (PMC5834615; doi:10.1038/s41467-018-03436-0)
Supplement: Supplementary file 2 — Peer Review File [file 41467_2018_3436_MOESM2_ESM.pdf]

Editorial Note: this manuscript has been previously reviewed at another journal that is not operating a transparent peer review scheme. This document only contains reviewer comments and rebuttal letters for versions considered at Nature Communications.

REVIEWERS' COMMENTS:

Reviewer #1 (Remarks to the Author):

The authors have properly considered all my hints in the revised version of the manuscript. Therefore, I propose now the publication of the paper in Nature Nanotechnology in its present form.
